# Arthrocolin B Impairs Adipogenesis via Delaying Cell Cycle Progression During the Mitotic Clonal Expansion Period

**DOI:** 10.3390/ijms26041474

**Published:** 2025-02-10

**Authors:** Guang Cao, Xuemei Liao, Shuang Zhao, Mengwen Li, Zhengyuan Xie, Jinglan Yang, Yanze Li, Zihao Zhu, Xiaoru Jin, Rui Huang, Ziyin Guo, Xuemei Niu, Xu Ji

**Affiliations:** 1 Key Laboratory of Medicinal Chemistry for Natural Resource, Ministry of Education, Yunnan Provincial Center for Research & Development of Natural Products, School of Pharmacy, Yunnan University, Kunming 650500, China; caoguang@ynu.edu.cn (G.C.); lxm20180206@163.com (X.L.); szhao79@foxmail.com (S.Z.); lmw10042000@163.com (M.L.); yangjinglan2022@163.com (J.Y.); 15981537137@163.com (Y.L.); z13951612718@163.com (Z.Z.); 18341959580@163.com (X.J.); h934315927@163.com (R.H.); 15033412998@163.com (Z.G.); 2 NHC Key Laboratory of Healthy Birth and Birth Defect Prevention in Western China, Yunnan Population and Family Planning Research Institute, Kunming 650021, China; xiezhengyuan11@163.com; 3 Laboratory for Conservation and Utilization of Bio-Resources, Key Laboratory for Microbial Resources of the Ministry of Education, Yunnan University, Kunming 650500, China

**Keywords:** arthrocolins, biosynthesis, adipogenesis, mitotic clonal expansion, cell cycle

## Abstract

Obesity and its related diseases severely threaten people’s health, causing persistently high morbidity and mortality worldwide. The abnormal proliferation and hypertrophy of adipocytes mediate the expansion of adipose tissue, which is the main cause of obesity-related diseases. Inhibition of cell proliferation during the mitotic clonal expansion (MCE) period of adipogenesis may be a promising strategy for preventing and treating obesity. Arthrocolins are a series of fluorescent dye-like complex xanthenes from engineered *Escherichia coli*, with potential anti-tumor and antifungal activities. However, the role and underlying mechanisms of these compounds in adipocyte differentiation remain unclear. In this study, we discovered that arthrocolin B, a member of the arthrocolin family, significantly impeded adipogenesis by preventing the accumulation of lipid droplets and triglycerides, as well as by downregulating the expression of key factors involved in adipogenesis, such as SREBP1, C/EBPβ, C/EBPδ, C/EBPα, PPARγ, and FABP4. Moreover, we revealed that this inhibition might be a consequence of cell cycle arrest during the MCE of adipocyte differentiation, most likely by modulating the p53, AKT, and ERK pathways, upregulating the expression of p21 and p27, and repressing the expression of CDK1, CDK4, Cyclin A2, Cyclin D1, and p-Rb. Additionally, arthrocolin B could promote the expression of CPT1A during adipocyte differentiation, implying its potential role in fatty acid oxidation. Overall, our research concludes that arthrocolin B has the ability to suppress the early stages of adipocyte differentiation mainly by modulating the signaling proteins involved in cell cycle progression. This work broadens our understanding of the function and mechanisms of arthrocolins in regulation of adipogenesis and might provide a potential lead compound for treating the obesity.

## 1. Introduction

Obesity has become a critical worldwide public health issue, posing a threat to people’s lives due to its increasing prevalence [1] and its links to a variety of serious health complications such as type 2 diabetes, heart disease, osteoarthritis, obstructive sleep apnea, and several types of cancer [2,3,4]. Adipose tissue (AT) is a versatile organ found throughout the body, possessing an almost unlimited capacity to expand during obesity [5]. Mammals possess two primary forms of adipose tissue (AT): white and brown. White adipose tissue (WAT) makes up the majority of the body’s total AT, typically surrounding major organs and blood vessels within the abdominal region and under the skin. In contrast, brown adipose tissue (BAT) comprises approximately 4.3% of an adult’s total fat mass and is predominantly situated in specific areas, such as the interscapular and supraclavicular regions [5,6]. WAT is crucial for the body’s energy balance, serving as the main site for energy storage after eating and as the source of free fatty acids during fasting [7]. WAT expansion is associated with the progression of many obesity and metabolic-related diseases [5,8,9]. The increase of adipocyte numbers (hyperplasia) and/or size (hypertrophy) is the main reason for causing WAT expansion, and the adipose tissue hyperplasia through the activation of progenitor cells is commonly regarded as a key mechanism for the expansion of adipose tissue [8,10]. Hence, it is a promising strategy to combat obesity by suppressing the adipogenesis.

Adipogenesis, the differentiation of preadipocytes into mature adipocytes, is a complex biological process involving coordinated changes in gene expression, cell shape, and hormone sensitivity [11,12]. Adipogenesis is a multi-stage process that encompasses three distinct phases. Initially, multipotent mesenchymal stem cells (MSCs) are directed towards the adipocyte lineage in response to specific physicochemical cues, transitioning into preadipocytes with minimal morphological alterations. The second stage is the mitotic clonal expansion (MCE), characterized by the reactivation of the cell cycle in previously quiescent cells, which then proceed through one or two cell cycles at the onset of differentiation. The final stage is terminal differentiation, during which there is a marked upregulation of various transcription factors and lipogenic genes, including the CCAAT/enhancer-binding proteins (C/EBPs), peroxisome proliferator-activated receptor gamma (PPARγ), sterol regulatory element-binding protein-1 (SREBP1), and fatty acid binding protein 4 (FABP4). This stage is also marked by morphological changes that facilitate the accumulation of lipids within the cells [13,14,15].

In the early phase of adipogenesis, mitotic clonal expansion plays a key role in driving the transformation of adipocytes precursors. However, the cell cycle control is essential for the MCE [14]. The cell cycle is fundamentally regulated by a set of cyclin proteins and cyclin-dependent kinases (CDKs), which form complexes and drive the cell through the different phases of the cell cycle when activated [16]. For instance, cyclin D1 binds to activate CDK4 and CDK6, while cyclin E binds to activate CDK2, which together for phosphorylation of the retinoblastoma protein (Rb), preventing it from binding and inhibiting the E2F transcription factor, allowing E2F to activate the transcription of proteins essential for the G1/S transition, thereby advancing the cell to the next stages of the cell cycle. Cyclin A forms complexes with CDK2 and CDK1, guiding the cell through the S and G2 phases. Upon the conclusion of the G2 phase, cyclin B-CDK1 complexes take the cell through mitosis (M phase) to complete the division cycle [16,17]. Besides that, some other key factors found to involve in the regulation of cell cycle progression. p53, a tumor-suppressor protein, responds to DNA damage signals during G1 phase, initiating transcription of CDK inhibitors, p21 and p27, which inhibit activation of the necessary CDKs of G1 to phosphorylate pRb, and therefore arresting the cells at the G1/S boundary [18]. Additionally, phosphatidylinositol 3-kinase/Akt and MEK/extracellular signal-regulated kinase (ERK) signaling pathways also participate in the cell cycle regulation. Inhibition of the phosphorylation of Akt or ERK will block transition of the G2/M phase and suppress tumorigenesis [19,20,21]. Several chemicals have been reported to suppress the adipogenesis through blockage of the progression of MCE by modulating the expression of cell cycle-related regulators, especially core CDKs and cyclins responsible for the cell cycle transition [10,22,23,24]. Accordingly, impairment of adipogenesis by delaying the progression of MCE of adipocytes may be a potential target for anti-obesity drugs development.

Xanthenes are an important class of heterocyclic aromatic compounds with diverse applications in pharmacology, industry, and synthesis. They include renowned fluorescent dyes like fluorescein, rhodamines, eosins, and the catalyst xantphos [25,26]. Arthrocolins are semi-naturally occurring fluorescent dye-like complex xanthenes embedded with indolyltriphenyl quaternary carbons, which were produced in an engineered *Escherichia coli* feeding with toluquinol or hydroquinones [27,28]. Recent studies show that arthrocolins A to C can strongly inhibit various human cancer cell lines, restore the antifungal activity of fluconazole against fluconazole-resistant *Candida albicans*, and work synergistically with fluconazole to lower its minimum inhibitory concentration. This also significantly improves the survival rates of 293T human cells and *Caenorhabditis elegans* infected with fluconazole-resistant *C. albicans* [27,29]. However, other pharmacological roles of arthrocolins are rarely known, especially their effects on adipogenesis. Herein, we found that arthrocolin B could hamper adipogenesis through delaying cell cycle progression of mitotic clonal expansion and elucidated the underlying mechanism. Our work enlarges the understanding of the pharmacologic functions of these novel biosynthetic fluorescein-like arthrocolins and provides a new potential therapeutic candidate for treating obesity.

## 2. Results

### 2.1. Arthrocolin B Suppresses the Differentiation of 3T3-L1 Preadipocytes into Adipocytes

**Figure 1:**
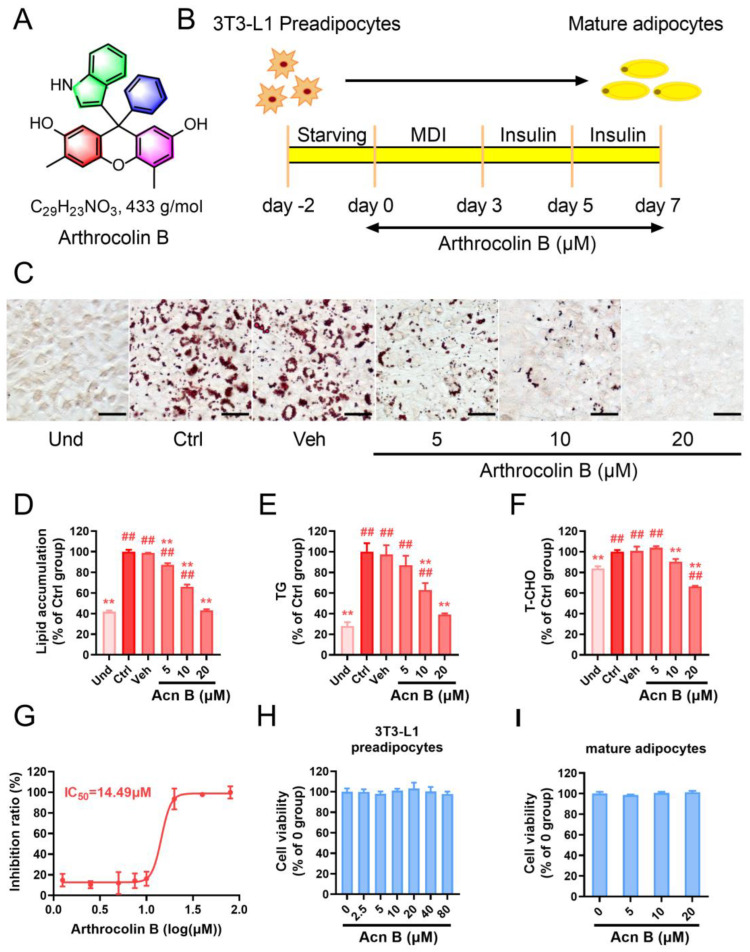
Arthrocolin B inhibits 3T3-L1 preadipocytes differentiation into mature adipocytes. (**A**) The chemical structure of arthrocolin B. (**B**) Schematic of the protocol for inducing 3T3-L1 preadipocytes to differentiate into mature adipocytes. Arthrocolin B was applied to 3T3-L1 preadipocytes for 7 days at concentrations ranging from 5 to 20 μM. Cells were harvested on day 7 for assessment of intracellular lipid levels. (**C**) Representative images of 3T3-L1 cells treated with various concentrations of arthrocolin B for 7 days and stained with Oil Red O. Scale bar = 50 μm. (**D**) Quantification of intracellular lipids by assessing Oil Red O staining intensity in the cells shown in panel C. (**E**) Normalized quantification of intracellular triglyceride (TG) content in the cells from panel C using a specific kit-based assay. (**F**) Intracellular total cholesterol (T-CHO) levels in the cells shown in panel C were determined using a specialized kit-based assay. (**G**) The IC_50_ value of arthrocolin B in inhibiting lipid accumulation. (**H**) 3T3-L1 preadipocytes were treated with arthrocolin B at concentrations ranging from 0 to 80 μM for 24 h, and their cell viability was evaluated using a Cell Counting Kit-8 (CCK-8) assay. (**I**) Cell viability of mature adipocytes treated with different doses of arthrocolin B during the entire differentiation stage (7 days) was assessed using a Cell Counting Kit-8 (CCK-8) assay. All values represent the mean ± SD (*n* ≥ 3) from three independent experiments. Significance is indicated as ^##^ *p* < 0.01 versus the Und group; ** *p* < 0.01 versus the Veh group. Und, undifferentiated preadipocytes; Ctrl, normally differentiated preadipocytes; Veh, normally differentiated preadipocytes treated with dimethyl sulfoxide (DMSO); Acn B, arthrocolin B; MDI, methylxanthine, dexamethasone, and insulin.

Arthrocolin B is a fluorescent dye-like complex xanthene produced in engineered *Escherichia coli* [27]. Its chemical structure is shown in Figure 1A. To investigate its effect on adipogenesis, we used the 3T3-L1 cell model. The differentiation of 3T3-L1 preadipocytes into mature adipocytes was induced as illustrated in Figure 1B, and lipid accumulation was assessed using Oil Red O staining. On day 7, differentiated 3T3-L1 cells exhibited significantly higher intracellular lipid droplet accumulation than undifferentiated cells (Figure 1C,D). However, when arthrocolin B was administered during differentiation, intracellular lipid accumulation was dose-dependently reduced. At a higher dose (20 μM), arthrocolin B-treated differentiated cells showed no significant difference in lipid accumulation compared to undifferentiated cells (Figure 1C,D). Similar reductions were observed in intracellular triglyceride (TG) content (Figure 1E) and total cholesterol (T-CHO) levels (Figure 1F). Using GraphPad Prism software, we determined the 50% inhibitory concentration (IC_50_) of arthrocolin B on intracellular lipid accumulation to be 14.49 μM (Figure 1G). Additionally, no cytotoxic effects were observed in either preadipocytes or mature adipocytes treated with arthrocolin B, as confirmed by the Cell Counting Kit-8 (CCK-8) assay (Figure 1H,I). These results indicate that arthrocolin B efficiently inhibits adipogenesis and the differentiation of 3T3-L1 preadipocytes.

### 2.2. Arthrocolin B Downregulates the Expression of Key Regulators in Adipocyte Differentiation

**Figure 2:**
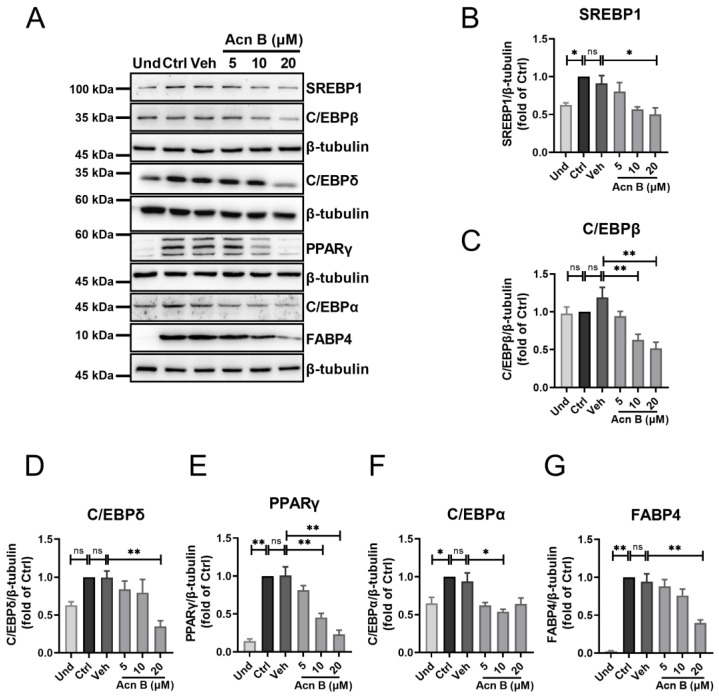
Arthrocolin B suppresses the key signaling pathway in adipocyte differentiation. (**A**) 3T3-L1 preadipocytes were treated with various doses of arthrocolin B for 7 days (the entire differentiation period). Cell lysates were then collected, and the expression levels of proteins SREBP1, C/EBPβ, C/EBPδ, PPARγ, C/EBPα, and FABP4 were analyzed by Western blotting. (**B**–**G**) Quantification of the bands from panel A is shown as relative protein expression levels, normalized to β-tubulin as an internal reference. Data represent the mean ± SEM from three independent experiments. Significance is presented as * *p* < 0.05, ** *p* < 0.01 between the indicated groups. ns: no significance. Und, undifferentiated preadipocytes; Ctrl, normally differentiated preadipocytes; Veh, normally differentiated preadipocytes treated with dimethyl sulfoxide (DMSO); Acn B, arthrocolin B.

Given the inhibitory effect of arthrocolin B on adipocyte differentiation observed previously, we further examined its impact on the expression of several key regulators involved in this process, including SREBP1, C/EBPβ, C/EBPδ, PPARγ, C/EBPα, and FABP4. As expected, the protein levels of SREBP1, PPARγ, C/EBPα, and FABP4 were significantly higher in differentiated 3T3-L1 preadipocytes compared to undifferentiated cells. However, treatment with arthrocolin B significantly reduced the protein expression of all these key regulators in differentiated 3T3-L1 preadipocytes (Figure 2A–G). These results suggest that arthrocolin B can effectively downregulate the expression of key regulators involved in adipocyte differentiation.

### 2.3. Arthrocolin B Regulates Adipogenesis in the Early Stage of Adipocyte Differentiation

**Figure 3:**
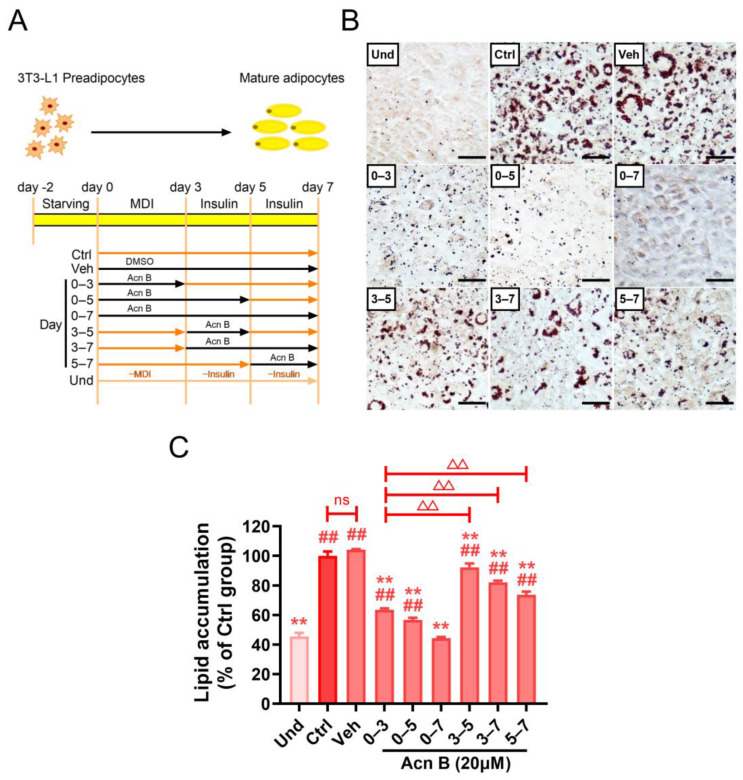
Arthrocolin B inhibits the early stage of adipocyte differentiation. (**A**) Schematic diagram showing the administration of arthrocolin B to 3T3-L1 cells at various stages of adipocyte differentiation. (**B**) Lipid droplet accumulation in 3T3-L1 preadipocytes treated with arthrocolin B at different stages, as shown in panel A, was assessed using Oil Red O staining. Scale bar = 50 μm. (**C**) Normalized quantification of lipid droplet accumulation in cells from panel B. Data are presented as the mean ± SEM (*n* ≥ 3) from three independent experiments. Significance is presented as ^##^ *p* < 0.01 versus the Und group; ** *p* < 0.01 versus the Veh group; ^△△^ *p* < 0.01 between the indicated groups. ns: no significance. Und, undifferentiated preadipocytes; Ctrl, normally differentiated preadipocytes; Veh, normally differentiated preadipocytes treated with dimethyl sulfoxide (DMSO); Acn B, arthrocolin B; MDI, methylxanthine, dexamethasone, and insulin.

To determine how arthrocolin B inhibits adipogenesis and differentiation, we divided the adipocyte differentiation process into three phases: early stage (day 0–3), intermediate stage (day 3–5), and terminal stage (day 5–7), based on previous reports [10,22]. We then treated 3T3-L1 preadipocytes with arthrocolin B at different phases to identify the most affected stage (Figure 3A). Compared to the vehicle control group (Veh), arthrocolin B treatment during days 0–3, 0–5, and 0–7 significantly reduced intracellular lipid accumulation. Treatments during days 3–5, 3–7, and 5–7 also reduced lipid accumulation, but the effects were weaker than those observed in the earlier phases. Specifically, lipid droplet accumulation was significantly lower in cells treated with arthrocolin B during the early stage (day 0–3) compared to those treated during the intermediate (day 3–5) and terminal stages (day 5–7) (Figure 3B,C). This suggests that arthrocolin B primarily exerts its inhibitory effects on adipogenesis during the early stage of differentiation.

### 2.4. Arthrocolin B Represses Cell Cycle Progression During MCE of the Early Stage of Adipogenesis

**Figure 4:**
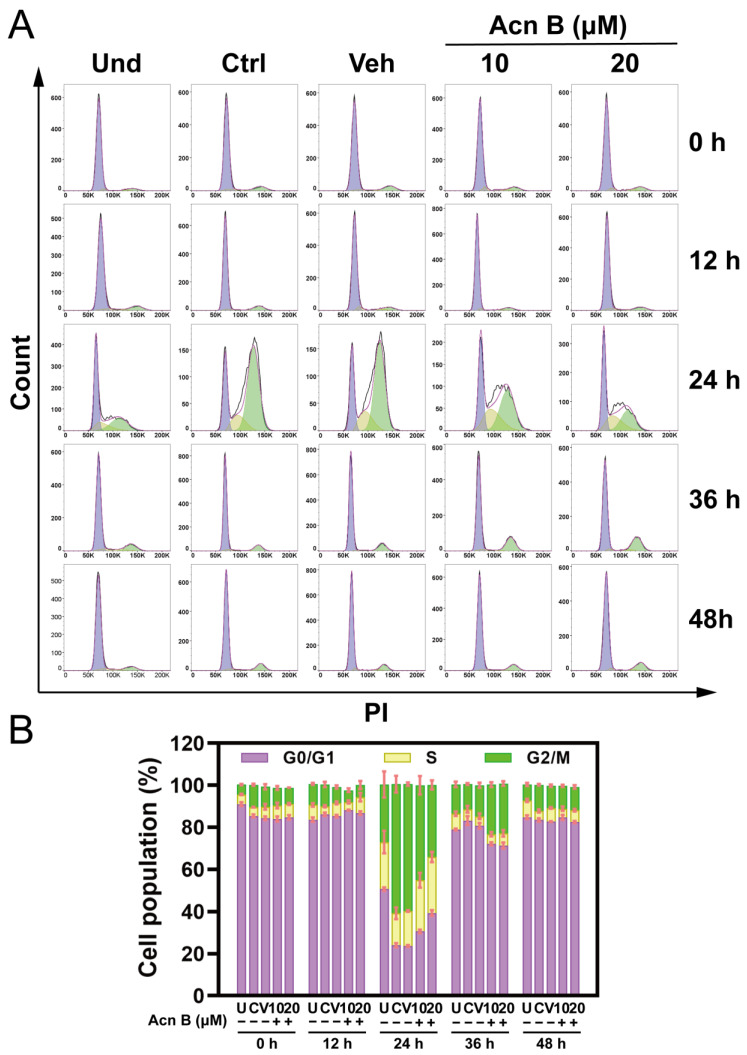
Arthrocolin B blocks cell cycle progression during MCE of adipogenesis. (**A**) Flow cytometry analysis of cells treated with 10 μM or 20 μM arthrocolin B for 0–48 h in the presence of differentiation agents. The colors light purple, yellow, and green in panel A indicate the proportions of cells in G0/G1, S, and G2/M phases, respectively. (**B**) Quantitative results of flow cytometry analysis from panel A. Values represent the percentages of cells in G0/G1, S, and G2/M phases. A total of 10,000 events were counted. Data are shown as the mean ± SEM (*n* ≥ 3) from three independent experiments. Und/U, undifferentiated preadipocytes; Ctrl/C, normally differentiated preadipocytes; Veh/V, normally differentiated preadipocytes treated with dimethyl sulfoxide (DMSO); Acn B, arthrocolin B.

Given that cell cycle variation during mitotic clonal expansion (MCE) is a key event in the early stage of adipogenesis, we hypothesized that the inhibitory effect of arthrocolin B on adipogenesis might be due to its impact on cell cycle progression during this early stage. To verify this, we examined the cell cycle of 3T3-L1 cells differentiated for 12 h, 24 h, 36 h, and 48 h, with cells treated simultaneously with 10 or 20 μM arthrocolin B. As expected, differentiation of 3T3-L1 preadipocytes for 24 h significantly increased the percentage of cells in the G2/M phase (from 27.35% to 61.28%) while decreasing the percentage in the G0/G1 phase (from 50.65% to 24.03%) and S phase (from 22.43% to 15.26%) compared to undifferentiated cells (Figure 4). This indicates that undifferentiated cells remained predominantly in the G0/G1 phase, whereas differentiated cells underwent typical cell cycle progression, transitioning from G0/G1 to S to G2/M within 24 h. However, treatment with 10 µM or 20 µM arthrocolin B for 24 h notably increased the percentage of cells in the G0/G1 phase (from 23.68% to 30.60% and 39.40%, respectively) and S phase (from 16.65% to 24.23% and 26.50%, respectively), while reducing the percentage in the G2/M phase (from 60.20% to 45.23% and 34.25%, respectively) compared to the vehicle group (Figure 4). In other words, arthrocolin B treatment diminished the differences in cell cycle distribution between undifferentiated and differentiated 3T3-L1 cells. Additionally, no significant differences in cell cycle distribution were observed at other time points during the 48-h differentiation period. Therefore, these results suggest that arthrocolin B suppresses adipogenesis by inhibiting cell cycle progression during the early stage of adipocyte differentiation, specifically during MCE.

### 2.5. Arthrocolin B Suppresses the Expression of Key Regulators Controlling the Cell Cycle Progression

**Figure 5:**
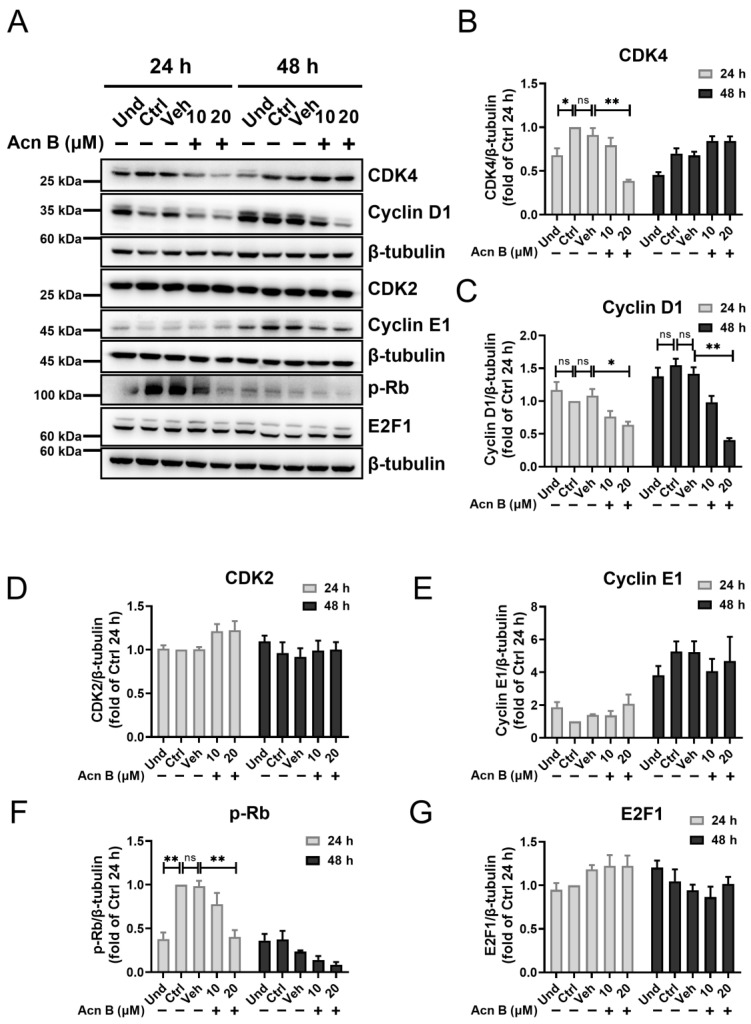
Arthrocolin B reduces the expression of key regulators associated with the G0/G1 phase. (**A**) 3T3-L1 preadipocytes were differentiated with MDI for 24 h or 48 h, and concurrently treated with 10 μM or 20 μM arthrocolin B. After that, cell lysates were collected, and the protein levels of CDK4, Cyclin D1, CDK2, Cyclin E1, p-Rb, and E2F1 were determined by Western blotting. (**B**–**G**) The intensity of the bands in panel A was quantified to determine the relative protein expression levels, with β-tubulin as an internal control. Data are shown as the mean ± SEM from three independent experiments. Significance is presented as * *p* < 0.05, ** *p* < 0.01 between the indicated groups. ns: no significance. Und, undifferentiated preadipocytes; Ctrl, normally differentiated preadipocytes; Veh, normally differentiated preadipocytes treated with dimethyl sulfoxide (DMSO); Acn B, arthrocolin B.

**Figure 6:**
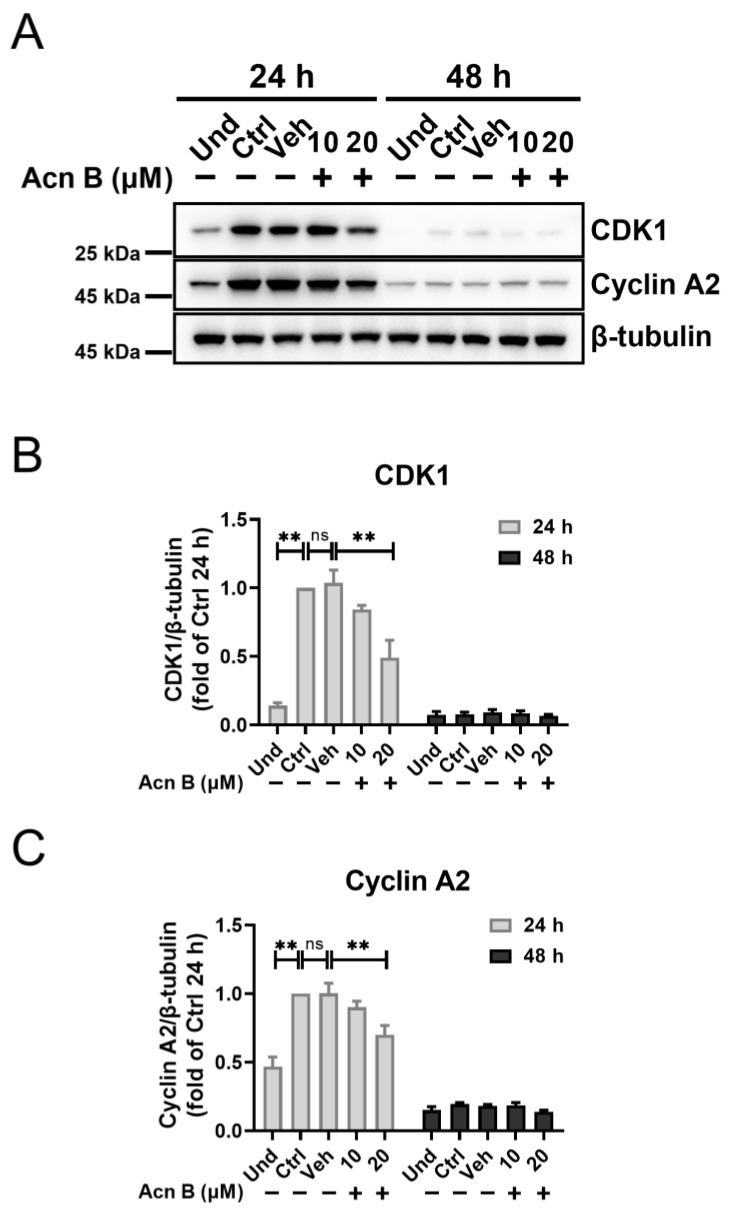
Arthrocolin B restrains the expression of key regulators associated with the S and G2/M phases. (**A**) 3T3-L1 preadipocytes were differentiated with MDI for 24 h or 48 h and concurrently treated with 10 μM or 20 μM arthrocolin B. After that, cell lysates were collected, and the protein levels of CDK1 and Cyclin A2 were analyzed by Western blotting. (**B**,**C**) The intensity of the bands in panel A was quantified to determine the relative protein expression levels, with β-tubulin as an internal control. Data are shown as the mean ± SEM from three independent experiments. Significance is presented as ** *p* < 0.01 between the indicated groups. ns: no significance. Und, undifferentiated preadipocytes; Ctrl, normally differentiated preadipocytes; Veh, normally differentiated preadipocytes treated with dimethyl sulfoxide (DMSO); Acn B, arthrocolin B.

To elucidate the mechanism underlying the cell cycle arrest mediated by arthrocolin B, we examined the expression changes of several key regulators involved in cell cycle progression, including CDK4, Cyclin D1, CDK2, Cyclin E1, CDK1, Cyclin A2, and Cyclin B1, in cells treated with arthrocolin B for 24 h or 48 h during adipocyte differentiation. The results showed that arthrocolin B treatment for 24 h significantly reduced the protein levels of CDK4 and Cyclin D1, which are crucial for the G1/S phase transition. However, the protein expression of CDK2 and Cyclin E1 was not significantly affected by arthrocolin B treatment at either 24 h or 48 h. Additionally, arthrocolin B treatment for 24 h dramatically decreased the protein levels of downstream p-Rb but had no effect on E2F1 expression (Figure 5A–G). Given the critical roles of CDK4/Cyclin D1 and p-Rb in regulating the G1/S phase transition, these findings suggest that arthrocolin B may cause cell cycle arrest in the G1/S phase during the early stages of 3T3-L1 preadipocyte differentiation. Furthermore, the expression of CDK1 and Cyclin A2 was also significantly reduced by arthrocolin B treatment at 24 h. However, we did not detect Cyclin B1 expression at this time point, likely due to its rapid degradation at the end of the G2 phase (Figure 6A–C). Since CDK2/Cyclin A2, CDK1/Cyclin A2, and CDK1/Cyclin B1 are essential for the G1/S, S/G2, and G2/M phase transitions, respectively, it is implied that arthrocolin B may obstruct these cell cycle phase transitions. In summary, these results suggest that arthrocolin B can hamper cell cycle progression by suppressing the expression of key cell cycle-related regulators.

### 2.6. Arthrocolin B Modulates the p53, ERK, and AKT Pathways During MCE

**Figure 7:**
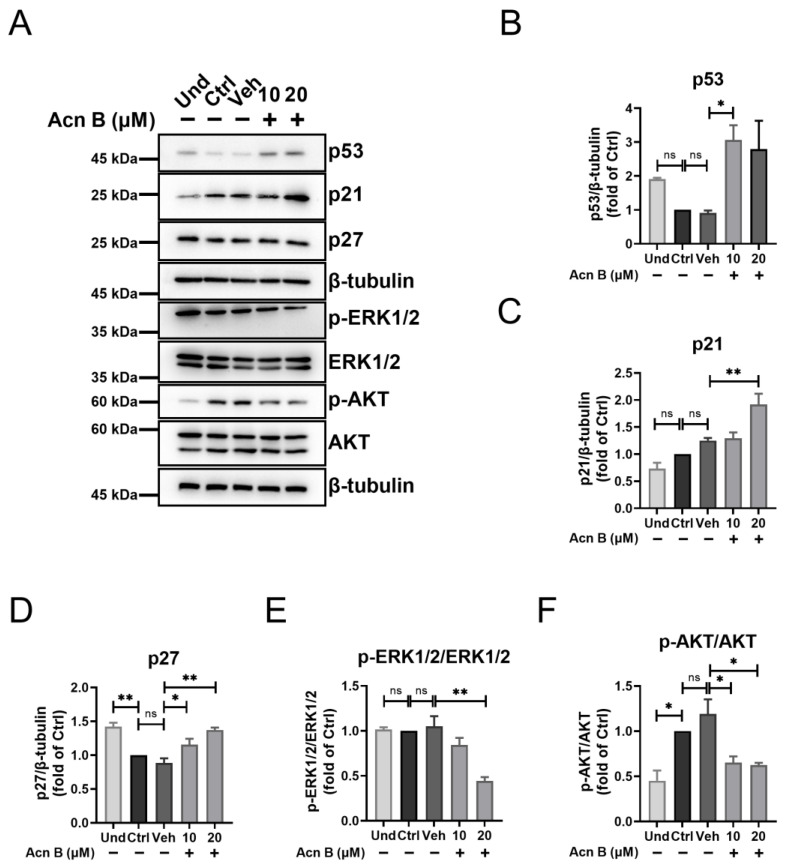
Arthrocolin B delays cell cycle progression during MCE of adipogenesis by regulating the p53, ERK, and AKT pathways. (**A**) 3T3-L1 preadipocytes were differentiated with MDI for 24 h and concurrently treated with 10 μM or 20 μM arthrocolin B. Subsequently, cell lysates were collected, and the protein levels of p53, p21, p27, p-ERK1/2, ERK1/2, p-AKT, and AKT were determined by Western blotting. (**B**–**F**) The intensity of the bands in panel A was quantified to determine the relative protein expression levels, with β-tubulin as an internal control. Data are shown as the mean ± SEM from three independent experiments. Significance is presented as * *p* < 0.05, ** *p* < 0.01 between the indicated groups. ns: no significance. Und, undifferentiated preadipocytes; Ctrl, normally differentiated preadipocytes; Veh, normally differentiated preadipocytes treated with dimethyl sulfoxide (DMSO); Acn B, arthrocolin B.

Next, we attempted to investigate the detailed regulatory mechanism of arthrocolin B in the process of MCE and adipocyte differentiation. 3T3-L1 preadipocytes were induced with MDI and treated with arthrocolin B for 24 h. The protein levels of p27, p21, and p53—which negatively regulate the cell cycle—were significantly increased by arthrocolin B treatment (Figure 7A–D). Additionally, the expressions of p-ERK1/2 and p-AKT were remarkably reduced by arthrocolin B treatment (Figure 7A,E,F). Given the roles of the p53-p21-RB, ERK, and AKT signaling pathways in cell cycle progression, arthrocolin B likely interferes with the cell cycle during adipocyte differentiation by modulating these pathways.

## 3. Discussion

Nowadays, obesity has emerged as a widespread epidemic, posing a significant threat to human health by impacting nearly every organ system. It has become a critical public health issue and is now classified among the most prevalent non-communicable diseases (NCDs) [30]. At present, the causes and mechanisms behind obesity are still not well understood. Currently, the most commonly used therapeutic strategies for obesity include lifestyle modification, calorie restriction combined with increased physical activity, pharmacotherapy, and bariatric surgery [31]. While lifestyle modification and physical fitness and exercise are hard to persist with, bariatric surgery is primarily reserved for patients with morbid obesity. Although anti-obesity drugs hold promise, their limited efficacy, coupled with potential side effects and drug interactions, underscores the ongoing need to discover new, effective, and safe anti-obesity ingredients [32,33]. For that purpose, it is necessary to identify new therapeutic targets for developing suitable anti-obesity drugs.

Inhibition of adipose tissue expansion seems a more credible strategy to combat obesity development than simply controlling the body weight [34]. White adipose tissue constitutes the largest proportion of whole-body adipose tissue, and adipocyte hyperplasia is generally considered the main contributor to its expansion [35]. Adipocyte hyperplasia, also known as adipogenesis, is primarily caused by the differentiation and maturation of preadipocytes. Therefore, it is a good strategy to find effective and safe drugs to treat obesity by preventing adipogenesis. Adipogenesis is a complex, multi-step process that is strictly regulated by a series of transcription factors, regulators, and signaling pathways [36]. Each step and related molecular regulators of adipogenesis are potential therapeutic targets. Currently, several small molecules—such as statins, fibrates, niacin, metformin, and liraglutide—and natural products, like delphinidin, genistein, guggulsterone, berberine, curcumin, etc., have been reported to affect adipogenesis [36,37,38,39]. However, our understanding of adipogenesis and its key regulators remains limited, and very few drugs have successfully translated to the clinic for fighting obesity by targeting adipogenesis. Accordingly, the search for new factors regulating adipogenesis and the development of novel anti-obesity drugs targeting adipogenesis remain urgent.

Arthrocolins represent a group of complex xanthene derivatives, similar to fluorescein, that are synthesized by genetically modified *Escherichia coli* strains when cultured with toluquinol or hydroquinones. These compounds are novel “unnatural” natural products and have not been reported in living organisms before [27], which has led to a lack of understanding about their biological functions and pharmacological activities. Although studies have shown that arthrocolins A to C can inhibit various human cancer cell lines, restore the antifungal activity of fluconazole against fluconazole-resistant *Candida albicans*, and synergize with fluconazole to protect 293T human cells and the nematode *Caenorhabditis elegans* from fluconazole-resistant *C. albicans* infection [27,29], this knowledge is still too limited and requires further and comprehensive investigation. Hence, in this study, we chose one of arthrocolins, specifically type B, to explore its function in adipogenesis.

Here, our results showed that arthrocolin B prominently inhibits the differentiation of 3T3-L1 preadipocytes to mature adipocytes, primarily during the early phase of adipocyte differentiation. Successful adipogenesis requires a postconfluent mitosis, known as mitotic clonal expansion (MCE), which occurs within the first 48 h after treatment with differentiation-inducing reagents. This process is crucial for DNA unwinding, allowing transcription factors to access regulatory elements in genes essential for the mature adipocyte phenotype [40,41]. Additionally, many natural products have been shown to suppress adipogenesis by regulating MCE [42,43]. Therefore, we hypothesized that arthrocolin B might inhibit adipogenesis by affecting MCE. As expected, our data showed that arthrocolin B treatment reduced the proportion of cells in the G2/M phase while increasing those in the G0/G1 and S phases at 24 h post-differentiation, indicating cell cycle arrest during the MCE period. To further validate this and elucidate the underlying mechanism, we examined the expression of key cell cycle regulators, including CDKs, cyclins, p-Rb, and E2F1, in undifferentiated or differentiated cells treated with or without arthrocolin B during the first 48 h of adipocyte differentiation. We found that the protein levels of CDK1, CDK4, Cyclin D1, Cyclin A2, and p-Rb decreased with increasing concentrations of arthrocolin B at 24 h. This likely explains the observed cell cycle arrest. Additionally, arthrocolin B upregulated the expression of p21 and p27, two CDK inhibitors, and their upstream transcription factor p53 during the early stage of differentiation. Since p53 activation is known to inhibit adipogenesis and enhance lipolysis [10,13,44], it is reasonable to propose that arthrocolin B affects MCE progression and adipocyte differentiation by activating the p53 pathway to regulate p21 and p27. Furthermore, arthrocolin B treatment significantly reduced the phosphorylation of Akt and ERK, which could cause cell cycle arrest at the G2/M transition [19,20,21]. Collectively, we speculate that arthrocolin B inhibits adipogenesis by suppressing MCE progression through modulating the p53, Akt, and ERK signaling pathways and repressing key CDKs and cyclins involved in cell cycle progression during early adipocyte differentiation.

Additionally, adipocyte differentiation is regulated by several adipogenic-specific genes, including peroxisome proliferator-activated receptor γ (PPARγ), CCAAT/enhancer-binding protein (C/EBP) family members, sterol regulatory element-binding protein-1 (SREBP1), and fatty acid binding protein (FABP) [15,41,45]. Moreover, adipocyte differentiation is heavily influenced by the interactions between cell cycle regulators and adipogenic transcription factors [10,13,22,46]. Therefore, in this study, we also evaluated the expression of several key regulators of adipogenesis, including SREBP1, C/EBPβ, C/EBPδ, PPARγ, C/EBPα, and FABP4. As expected, most of these regulators were upregulated by the end of the differentiation phase. However, arthrocolin B treatment led to the downregulation of all these regulators, further supporting its role in inhibiting adipogenesis.

**Figure 8:**
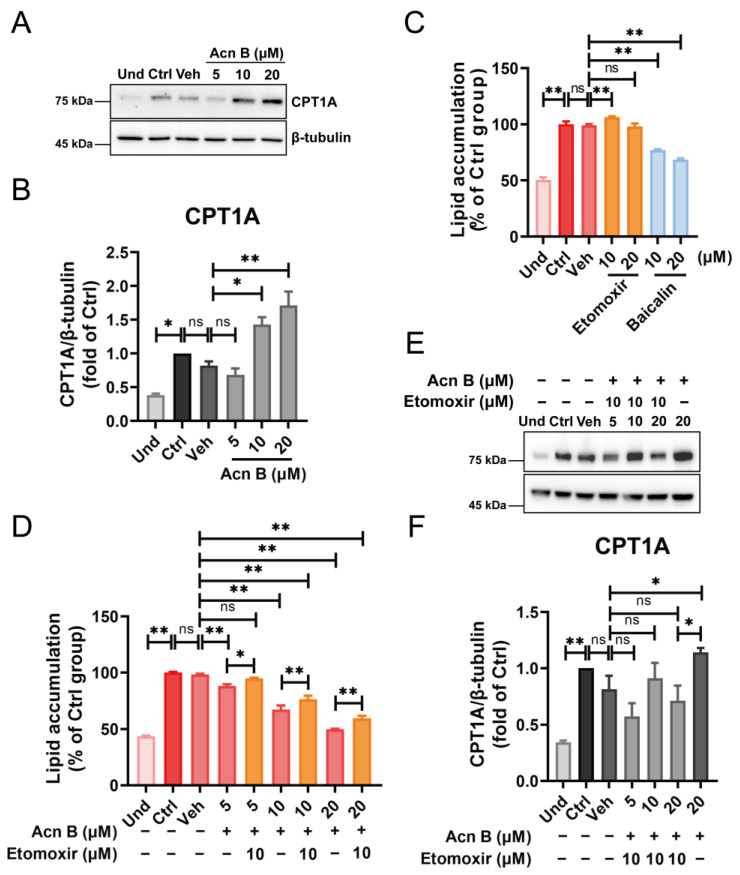
Arthrocolin B inhibits adipogenesis by promoting CPT1A expression. (**A**) 3T3-L1 preadipocytes were treated with various concentrations of arthrocolin B for 7 days (the entire differentiation period). Then, cell lysates were collected, and the protein levels of CPT1A were determined by Western blotting. (**B**) The intensity of the bands in panel A was quantified to determine the relative protein expression levels, with β-tubulin as an internal control. (**C**,**D**) 3T3-L1 preadipocytes were treated for 7 days with either 10 μM or 20 μM Etomoxir, baicalin, or various concentrations of arthrocolin B (with or without 10 μM Etomoxir). After treatment, intracellular lipid levels were assessed by Oil Red O staining and absorbance measurement at 510 nm. (**E**) 3T3-L1 preadipocytes were treated with various concentrations of arthrocolin B (with or without 10 μM Etomoxir) for 7 days. Then, cell lysates were collected, and CPT1A protein levels were determined by Western blotting. (**F**) The intensity of the bands in panel E was quantified to determine the relative protein expression levels, with β-tubulin as an internal control. Data are shown as the mean ± SD from three independent experiments. Significance is presented as * *p* < 0.05, ** *p* < 0.01 between the indicated groups. ns: no significance. Und, undifferentiated preadipocytes; Ctrl, normally differentiated preadipocytes; Veh, normally differentiated preadipocytes treated with dimethyl sulfoxide (DMSO); Acn B, arthrocolin B.

Currently, several molecules with inhibitory effects on the MCE phase of adipogenesis have been identified, including small molecules, natural products, specific proteins, and microRNAs. These inhibitors generally act by suppressing the expression of key adipogenic regulators such as C/EBPs, PPARγ, and FABP4, and by inhibiting core CDKs/Cyclins that drive cell cycle progression, leading to cell cycle arrest [47,48,49,50,51,52,53,54]. However, the upstream signaling pathways impacted vary among different MCE inhibitors. For example, hydroxycitric acid (HCA) inhibits the RPS6KA1/FoxO1 signaling axis, while isoeugenol attenuates the AKT, ERK, and MAPK pathways. Other examples include lactucin, which downregulates the JAK2/STAT3 signaling pathway, and ezetimibe, which regulates the AMPK-mTORC1 pathway [48,49,50,51]. The commonality among these MCE inhibitors is consistent with the effects of arthrocolin B, which also inhibits the AKT and ERK pathways. However, unlike most MCE inhibitors, arthrocolin B gradually enhances the suppression of intracellular lipid accumulation as differentiation progresses, suggesting it may also function in the middle and late phases of adipogenesis. Interestingly, we found that arthrocolin B promotes the expression of CPT1A (carnitine palmitoyltransferase-1A) during adipocyte differentiation (Figure 8A,B). Activation of CPT1A by its activator baicalin inhibits the adipocyte differentiation (Figure 8C), while inhibition of CPT1A by its specific inhibitor Etomoxir partially rescues the inhibitory effect of arthrocolin B on adipogenesis, with a reduction in CPT1A expression (Figure 8D–F). These results suggest that CPT1A is involved in regulating adipogenesis and may be a potential target of arthrocolin B. CPT1A is responsible for catalyzing the rate-limiting step in the carnitine shuttle, essential for mitochondrial beta-oxidation of long-chain fatty acids [55]. Given its critical role in fatty acid oxidation and triglyceride metabolism [55,56], we speculate that the influence of arthrocolin B on adipogenesis, especially in the middle and late phases, is due to its upregulation of CPT1A expression, which subsequently facilitates fat metabolism and attenuates intracellular lipid accumulation.

Additionally, our study still has some limitations. For example, the impact and mechanisms of arthrocolin B on metabolism, especially lipolysis, warrant thorough investigation. Furthermore, the in vivo anti-obesity effects of arthrocolin B need further determination. Additionally, the anti-adipogenesis effects and underlying mechanisms of the other two arthrocolins (A and C) require examination and elucidation in future studies.

In conclusion, arthrocolin B, a complex xanthene with fluorescent dye-like properties synthesized by an engineered *Escherichia coli*, can suppress the early stage of adipogenesis by delaying the MCE progression through the regulation of signaling proteins involved in cell cycle progression.

## 4. Materials and Methods

### 4.1. Chemicals and Reagents

Arthrocolin B was donated by Professor Xuemei Niu. Oil red O powder, 3-isobutyl-1-methylxanthine (IBMX), and dexamethasone (DEX) were purchased from Sigma (St. Louis, MO, USA). Total cholesterol (T-CHO) and triglyceride (TG) assay kits were obtained from Nanjing Jiancheng Bioengineering Institute (Nanjing, China). Cell Counting Kit-8 (CCK-8) and the enhanced chemiluminescence (ECL) reaction kit were purchased from Proteintech (Rosemont, IL, USA). Insulin from bovine pancreas and PI were obtained from Solarbio (Beijing, China). Pierce bicinchoninic acid (BCA) protein assay kit was purchased from Thermo Fisher Scientific (Waltham, MA, USA). Rabbit polyclonal antibodies against PPAR gamma, C/EBPα, FABP4, ERK1/2, phospho-ERK1/2 (Thr202/Tyr204), phospho-Rb (Ser807/811), p27, and β-tubulin were purchased from Proteintech (Rosemont, IL, USA). Rabbit monoclonal antibodies against Cyclin D1, Cyclin A2, phospho-AKT (Ser473), AKT, p53, p21, and CDK1 were purchased from Hua’an Biotechnology (Hangzhou, China). Rabbit polyclonal antibodies against CDK2, CDK4, and Cyclin E1, and rabbit monoclonal antibody against C/EBPδ were purchased from ABclonal Technology (Wuhan, China). Rabbit polyclonal antibody against C/EBPβ was obtained from Cell Signaling Technology (Danvers, MA, USA). Rabbit monoclonal antibody against E2F1 was purchased from Boster Biological Technology (Wuhan, China). Mouse monoclonal antibody against SREBP-1 was purchased from Santa Cruz Biotechnology (Dallas, TX, USA). Mouse monoclonal antibody against CPT1A was obtained from Abcam (Cambridge, UK). Horseradish peroxidase (HRP)-conjugated anti-rabbit IgG secondary antibody and HRP-conjugated anti-mouse IgG secondary antibody were purchased from Santa Cruz Biotechnology (Dallas, TX, USA).

### 4.2. Cell Culture, Differentiation, and Treatment

Mouse 3T3-L1 preadipocytes were purchased from the American Type Culture Collection (ATCC) (Manassas, VA, USA). The cells were cultured in Dulbecco’s modified Eagle medium (DMEM) supplemented with 10% newborn calf serum and 1% penicillin/streptomycin at 37 °C in a 5% CO_2_ atmosphere. For differentiation, once the cells reached 100% confluence, they were incubated for an additional two days (marked as day 0). The cells were then treated with a differentiation-inducing agent mixture (MDI) containing 500 μM IBMX, 0.25 μM dexamethasone, and 10 μg/mL insulin for three days. On day 3, the medium was replaced with DMEM containing 10% fetal bovine serum (FBS), 1% penicillin/streptomycin, and 10 μg/mL insulin for another five days (until day 7). Arthrocolin B was dissolved in DMSO, stored at −20 °C, and added to the medium. Cells were exposed to either arthrocolin B-supplemented medium or a control medium containing an equivalent volume of DMSO throughout the differentiation process.

### 4.3. Oil Red O Staining

Intracellular lipid accumulation was evaluated by Oil red O staining. Briefly, at the end of adipocyte differentiation, 3T3-L1 cells were washed three times with phosphate-buffered saline (PBS), fixed with 10% formaldehyde for 30 min at room temperature, and dehydrated with 60% isopropanol for 30 s. The cells were then stained with a filtered Oil red O working solution for 10 min at room temperature, rinsed four times with distilled water, and imaged using an optical microscope (Olympus, Tokyo, Japan) equipped with a digital camera. The Oil red O-stained lipid droplets were eluted with 100% isopropanol and quantified using a spectrophotometer (SpectraMax M2, Molecular Devices, San Jose, CA, USA) at 510 nm.

### 4.4. TG and T-CHO Content Assays

The levels of intracellular triglycerides (TG) and total cholesterol (T-CHO) in mature adipocytes were measured using specific assay kits. The cell suspension was collected, centrifuged with PBS at 500× *g* for 3 min, and the pellet was lysed with 1% Triton X-100 for 30 min. The TG and T-CHO levels were then determined using a microplate reader (SpectraMax M2, Molecular Devices, San Jose, CA, USA) at 500 nm. Calibration curves were generated by serial dilution of cholesterol and triglyceride standards.

### 4.5. Cell Viability Assay (By CCK-8)

3T3-L1 preadipocytes were seeded at a density of 1 × 10^5^ cells/well in 96-well plates. After 24 h of culture, the cells were treated with arthrocolin B at concentrations ranging from 0 to 80 μM for 48 h. Cell viability was then assessed using a Cell Counting Kit-8 (CCK-8) according to the manufacturer’s instructions. To analyze the effect of arthrocolin B on mature adipocytes, 3T3-L1 preadipocytes were seeded at the same density, induced to differentiate into mature adipocytes using MDI for 7 days, and treated with arthrocolin B at concentrations ranging from 0 to 20 μM throughout the differentiation process. The viability of mature adipocytes was subsequently measured using CCK-8.

### 4.6. Cell Cycle Assay

To analyze the cell cycle of 3T3-L1 preadipocytes during early adipocyte differentiation, 3T3-L1 cells were starved for two days, then incubated with MDI and treated with or without 10 μM or 20 μM arthrocolin B for 12, 24, 36, and 48 h. Cells were harvested, washed three times with PBS, and fixed in 70% cold ethanol at −20 °C overnight. The next day, cells were centrifuged to remove ethanol, washed twice with pre-cooled PBS, incubated with 10 μg/mL RNase A for 30 min at 37 °C, and stained with 25 μg/mL PI solution for 10 min at room temperature. At least 10,000 cells per sample were analyzed using a BD FACS Calibur™ Flow Cytometer (BD Biosciences, Franklin Lakes, NJ, USA), and the data were processed with Flowjo 10.5.3 software to determine the distribution of cells in the G0/G1, S, and G2/M phases of the cell cycle.

### 4.7. Protein Extraction and Western Blot Procedure

Cells treated with or without arthrocolin B were washed twice with cold PBS to remove residual medium. The cells were lysed with RIPA buffer containing protease and phosphatase inhibitors. Lysates were collected by scraping cells from petri dishes, incubated on ice for 10 min, and then centrifuged at 10,000× *g* for 10 min at 4 °C to obtain the supernatant. Total protein concentration was measured using a Pierce BCA protein assay kit. The protein samples were denatured by heating at 100 °C for 10 min. Equal amounts of protein were separated by 10% SDS-PAGE and transferred to 0.45 μm PVDF membranes (Merck Millipore, Burlington, VT, USA). The membranes were blocked with 5% skim milk in TBST for 2 h and incubated with primary antibodies overnight at 4 °C. After washing three times with TBST, the membranes were incubated with HRP-conjugated secondary antibodies for 1 h at room temperature. Protein bands were visualized using enhanced chemiluminescence reagent and imaged with the Tanon 5200 Multi imaging system (Shanghai, China).

### 4.8. Statistical Analysis

All experiments were repeated three times. The intensity of bands in the Western blot images was measured using Image J (Fiji) software. Statistical analysis was performed using GraphPad Prism 9.5 software, and the statistical significance of the differences between the indicated groups were determined by one-way analysis of variance (ANOVA) with Tukey’s multiple comparison test. All data are presented as the mean ± standard deviation (mean ± SD) or mean ± standard error (mean ± SEM) from three independent experiments. The sample size (*n*) in each experiment is mentioned in the figure legends. *p* < 0.05 was considered statistically significant.

## Data Availability

The raw data supporting the conclusions of this article will be made available by the authors on request.
